# Reducing Racial Disparities in Hypertension Control Using a Multicomponent, Equity-Centered Approach

**DOI:** 10.1177/24731242251371424

**Published:** 2025-08-27

**Authors:** Susan Mwikali Kioko, Christina Council, Cecilia Tomori

**Affiliations:** ^1^ Rockville Internal Medicine Group, Rockville, Maryland, USA.; ^2^ One Medical Group, Potomac, Maryland, USA.; ^3^ Johns Hopkins School of Nursing, Johns Hopkins University, Baltimore, Maryland, USA.; ^4^ Department of Population, Family and Reproductive Health, Johns Hopkins Bloomberg School of Public Health, Johns Hopkins University, Baltimore, Maryland, USA.

**Keywords:** hypertension, African American, Black American, health equity, multicomponent intervention, primary care

## Abstract

**Introduction:**

Black Americans have the highest prevalence of hypertension among all racial or ethnic groups in the United States. They are 40% more likely to have uncontrolled blood pressure (BP) and are five times more likely to die from hypertension compared with non-Hispanic Whites. Experiences of discrimination in health care, clinician and institutional bias, and socioeconomic and environmental inequities driven by structural racism contribute to uncontrolled hypertension in this population. Multilevel, multicomponent interventions have effectively improved BP control among Black Americans but remain inadequately implemented in the clinical setting. An integrated nursing/public health quality improvement study was designed to address this gap between evidence and integration into clinical practice.

**Methods:**

Using a one group pre/posttest design, we examined the effect of an innovative, evidence-based 12-week intervention on BP among Black Americans with uncontrolled hypertension aged 18 and older in the primary care setting. Intervention components included remote BP monitoring, weekly phone coaching with culturally congruent care, medication intensification, and a standardized hypertension protocol.

**Results:**

The average age of the participants (*n* = 35) was 64 years, and two thirds (*n* = 23) were female (66%). The mean difference in systolic BP from pre to postintervention decreased significantly (M = 23, standard deviation [SD] = 14.0), *t(34)* = –9.7, *p* < 0.001). A significant reduction in the mean difference in diastolic BP from pre to postintervention was also observed (M = 11, SD = 11.8), *t(34)* = –5.5, *p* < 0.001). At 12 weeks, 87% of participants had achieved BP control. The intervention also improved medication adherence and hypertension knowledge (*p* < 0.001).

**Conclusion:**

A multicomponent, culturally congruent quality improvement intervention may effectively improve BP among Black Americans.

**Health Equity Implications:**

Scaled up implementation of equity-centered, culturally congruent approaches is needed to reduce racial disparities in hypertension control.

## Introduction

In the United States, Black Americans have the highest prevalence of hypertension among all racial or ethnic groups.^1^ They are 40% more likely to have uncontrolled blood pressure (BP) compared with non-Hispanic Whites despite comparable hypertension awareness and treatment rates.^2,9^ Black Americans develop hypertension at an earlier age, at greater severity, and with faster disease progression than their counterparts.^10^ They are five times more likely to die from hypertension than non-Hispanic Whites,^3,4^ experiencing the highest hypertension-related morbidity and premature mortality among all racial/ethnic groups.^11^

Multiple factors rooted in systemic racism are responsible for the racial disparity in hypertension prevalence and control. Individual-level factors, such as treatment adherence, are informed by health beliefs, health literacy, and experiences of discrimination in health care.^7,11^ Practice and provider-level factors, including implicit bias, influence clinical decision-making and treatment intensification.^3,7,12^ Overarching factors such as structural racism shape the socioeconomic and environmental milieu that structure the risk for hypertension and poor BP control, including the affordability of medication, availability and cost of healthy food, access to recreational spaces, and exposure to psychosocial and behavioral risk factors that affect hypertension control.^3,5–8^

Advancing health equity is a national priority outlined in the Framework for Health Equity^13^ and the Healthy People 2030 Framework.^14^ Equity-based recommendations include evidence-based quality improvement initiatives that address social risk factors and gaps in health outcomes,^13^ targeted interventions to improve BP control among racial/ethnic minorities,^4,14^ and investing in populations who experience significant disparities in health.^13^ Previous studies have successfully utilized multicomponent interventions that combine BP monitoring, drug therapy, hypertension lifestyle counseling, and care coordination with community health workers (CHWs) and pharmacists for hypertension management among Black Americans.^15–21^ These interventions, however, remain inadequately implemented, with most primary care settings emphasizing drug therapy without systems of support for lifestyle counseling and follow-up. Dominant approaches often fail to provide culturally congruent care or address health-related social needs when delivering hypertension care, with lifestyle counseling generally limited to advice to eat better or exercise more without practical, culturally relevant guidance or support tailored to the lived experience.^15,22^ This quality improvement project aimed to address this gap between evidence and translation into clinical practice and to shed light on the pragmatic value of implementing a more comprehensive intervention for hypertension management.

Utilizing the social ecological framework,^23^ this quality improvement project was undertaken to develop, implement, and evaluate a 12-week multicomponent intervention targeting barriers to BP control among hypertensive Black Americans in the primary care setting at different levels of influence. Drawing on best practice from existing literature, individual barriers to hypertension management including hypertension knowledge and medication adherence were addressed through weekly hypertension lifestyle coaching using culturally congruent care.^3,6,17,20,24^ Practice and provider-level components addressed provider adherence to treatment guidelines and therapeutic inertia through standardized hypertension care processes and decision support tools.^16,18^ Remote patient monitoring and utilization of diverse communication platforms expanded clinical services increasing access to care.^9,15,19^

Our aims were to evaluate the impact of the intervention on BP, medication adherence, and hypertension knowledge. The goal of this intervention was to provide a potential model for implementing innovative, effective, and equity-centered approaches into clinical practice and to inform policies on best practices for eliminating health inequities in BP control.

## Methods

### Design

This quality improvement project was designed as a one-group pre/posttest evaluating the effectiveness of a 12-week multicomponent intervention on BP among hypertensive Black Americans in the primary care setting and undertaken as the first author’s DNP/MPH Scholarly Project at Johns Hopkins University. Components of the intervention included daily home BP monitoring (HBPM) with remote patient monitoring (RPM); 15-min weekly phone coaching on hypertension self-management and lifestyle; 15-min biweekly medication management based on 2-week average BP value of >140/90; and a standardized hypertension protocol. Patient education was based on the American Heart Association guidelines. Participants were given remote BP monitors to use at home during the study. The primary outcome measure was BP change from enrollment using an automatic BP monitor.

### Setting and sample

The project was implemented at a primary care and specialty practice in the D.C. Metro area from September 2023 through December 2023. A sample of 35 participants was recruited through provider referral and telephone outreach. Participants were eligible for the program if: (1) self-identified as African American or Black; (2) aged 18 and older; (3) diagnosed with hypertension ICD code: I10 in electronic health record (EHR); (4) uncontrolled hypertension as defined by BP >140/90 or >130/80 mmHg with type 2 diabetes; (5) English speaking; (6) active phone line; and (7) established primary care patient. Patients diagnosed with chronic kidney disease, congestive heart failure, active malignancy, or pregnancy were excluded from the study. The study was approved by the Johns Hopkins School of Nursing Ethical Review Committee.

### Intervention procedures

Preimplementation, a multidisciplinary team developed a standardized protocol for managing elevated BP when identified during an office visit (see Supplementary Appendix). The protocol reflected best practice recommendations for hypertension management. During the enrollment period, informed consent and baseline data were collected, and participants were enrolled into remote BP monitoring. Patients were instructed on BP monitoring following standard clinical guidelines. They were instructed to measure their BP at the same time every day and to check two readings 1–2 min apart. Proper measurement technique and wireless transmission of BP measurement were verified at the time of enrollment, with the first measurement constituting baseline systolic BP (SBP) and diastolic BP (DBP). Questionnaires were completed through self-report or a structured interview pre/postintervention and examined for completeness. BP flow charts were reviewed daily, and patients outreached as needed for SBP <90 or >160 and DBP <50 or >100. Patients received weekly phone coaching on hypertension self-management and lifestyle, including medication adherence, low-sodium diet, physical activity, weight management, alcohol limitation, smoking cessation, stress management, and communication training. Phone coaching was delivered by the student investigator with specific recommendations tailored to individual needs and abilities. Patients received text message reminders for phone coaching sessions, and two attempts were made to connect with the patient for phone coaching. If the patient was unable to attend the session, it was rescheduled within the same week or deferred to the following week. BP control was assessed biweekly, and treatment intensified based on a 2-week average BP value of >140/90. All patient encounters were recorded in the EHR and sent to the primary care provider for review. Fidelity throughout the study was ensured by regular meetings with the implementation team.

### Measures/instruments

Demographic data were extracted from the EHR. Pre/postintervention BP was obtained from the remote BP monitor log through the measurement of SBP and DBP using an automatic wireless BP machine. Hypertension knowledge was measured using the Hypertension Knowledge Test, which has a Cronbach’s *α* of 0.92, indicating high reliability. It is a self-reported, 20-item questionnaire with four domains: creating awareness of the disease condition, lifestyle modifications, dietary regimen, and exercise regulation. A score of 80% indicates adequate knowledge.^25^ Medication adherence was assessed through the Morisky Medication Adherence Scale (MMAS-8). The MMAS-8 has acceptable pooled estimates of reliability in meta-analysis when used in hypertension and is widely used to assess medication adherence in chronic conditions.^24^ It is a self-reported questionnaire consisting of eight items that assess forgetfulness, symptom severity, and emotional and situational aspects of medication adherence.^24^ Higher scores indicate higher medication adherence. Feasibility was assessed through adherence to daily BP monitoring, weekly attendance to phone coaching sessions, program completion, and provider adherence to the hypertension protocol.

### Data analysis

Data were analyzed using the Statistical Package for the Social Sciences version 29.^26^ Descriptive statistics were used to analyze characteristics of the sample population, adherence to daily BP measurement, phone session attendance rate, program completion, and adherence to the hypertension protocol. Although nonparametric tests were considered, the mean difference in pre/postintervention SBP and DBP, medication adherence, and hypertension knowledge test scores were analyzed using a paired *t* test due to the approximately normal distribution of the data.

## Results

### Demographics

All 35 participants in this study were Black American and most were female (65.7%). Their age ranged from 34 to 84 years with a mean age of 64 years (±13.7). Of the 35 participants enrolled, 40% (*n* = 14) had comorbid type 2 diabetes and 85.7% (*n* = 30) had uncontrolled hypertension as defined by BP >140/90 or >130/80 mmHg with comorbid type 2 diabetes. All the participants were on antihypertensive medication at enrollment. The average body mass index in the study sample was 32.8 (±7.1). Thirty-four participants (97.1%) were insured at the start of the study. Most participants were not working (54.3%), with 48.6% (*n* = 17) retired and 5.7% (*n* = 2) unemployed. Data on education level and number of years with hypertension diagnosis were incomplete in the EHR and therefore not collected (see Table 1).

**Table 1.** Baseline Characteristics of Study Participants, *N* = 35

Study characteristics	*N*	%
Age >65 years	20	57
Gender		
Women	23	66
Men	12	34
Race (Black American)	35	100
Blood pressure		
Controlled	5	14
Uncontrolled	30	86
Comorbidity		
Diabetes mellitus	14	40
Obesity (BMI > 30)	20	57
Insurance status		
Medicare	20	57
Other	14	40
None	1	3
Employment		
Employed	16	46
Unemployed	2	5
Retired	17	49

BMI, body mass index.

### Systolic and diastolic blood pressure

**FIG. 1.**
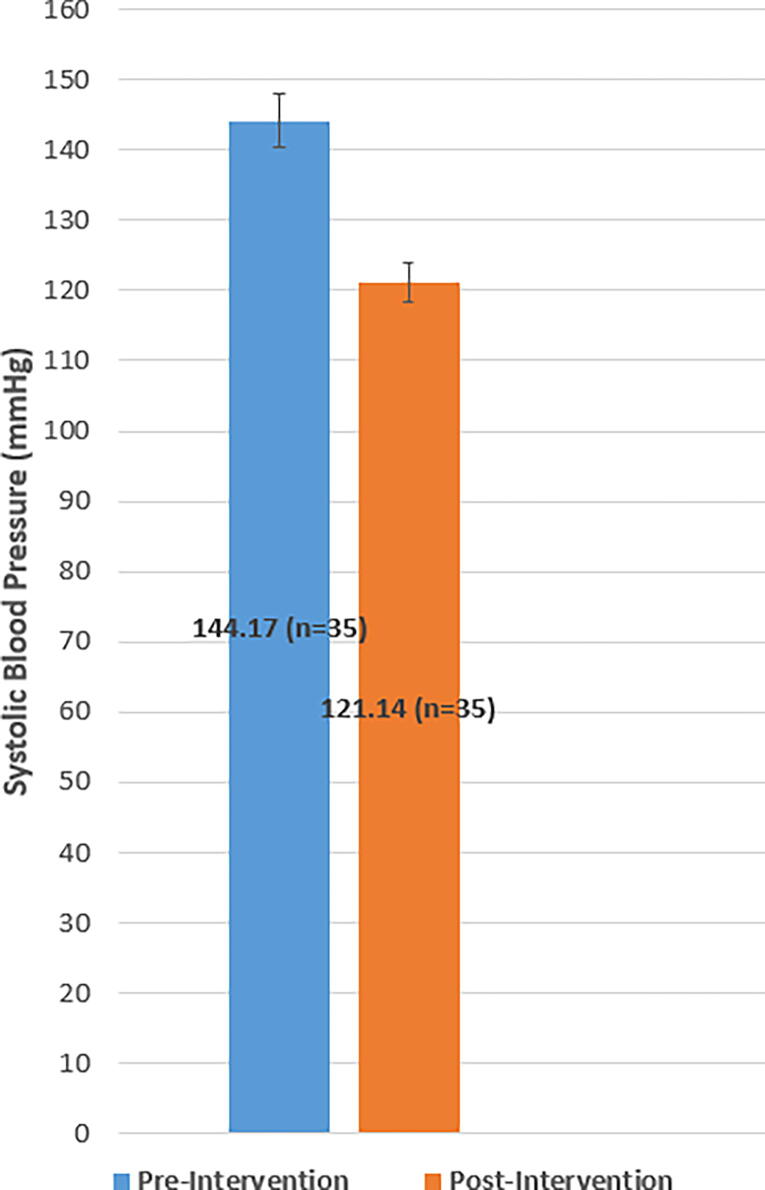
Mean systolic blood pressure (mmHg) at baseline and postintervention. Preintervention systolic blood pressure averaged 144 mmHg ± 11.8, 95% CI: [140.1, 147.9] and postintervention averaged 121 mmHg ± 8.7, 95% CI: [118.1, 123.9]. Error bars represent 95% CIs. CI, confidence interval.

**FIG. 2.**
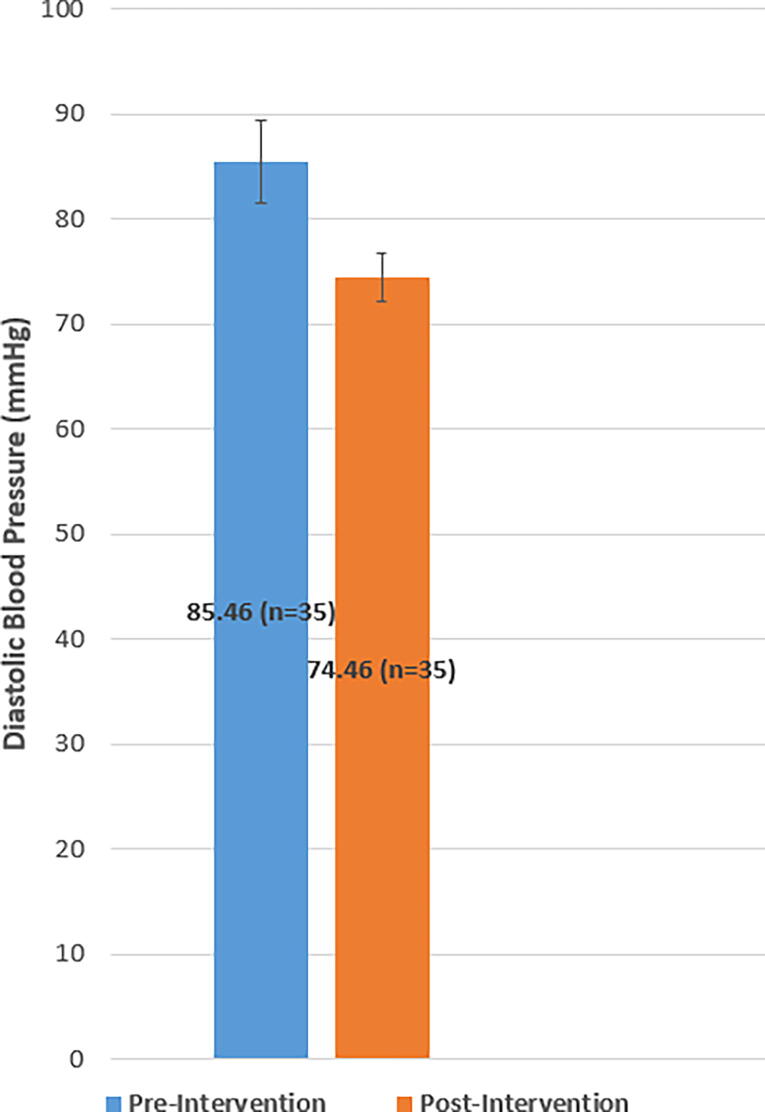
Mean diastolic blood pressure (mmHg) at baseline and postintervention. Preintervention diastolic blood pressure averaged 85 mmHg ± 12.1, 95% CI: [80.9, 89.0] and postintervention averaged 74 mmHg ± 6.8, 95% CI: [71.7, 76.3]. Error bars represent 95% CIs. CI, confidence interval.

The average SBP preintervention was 144 mmHg ± 11.8, 95% confidence interval (CI): [140.1, 147.9], and the average SBP postintervention was 121 mmHg ± 8.7, 95% CI: [118.1, 123.9]. The mean difference in SBP from pre to postintervention decreased significantly (M = 23, standard deviation [SD] = 14.0, 95% CI: [18.4, 27.6]) *t(34)* = –9.7, *p* < 0.001 (see Fig. 1). The effect size as measured by Cohen’s *d* was 2.22, indicating a very large effect size. The average DBP preintervention was 85 mmHg ± 12.1, 95% CI: [80.9, 89.0], and the average DBP postintervention was 74 mmHg ± 6.8, 95% CI: [71.7, 76.3]. The mean difference in DBP from pre to postintervention decreased significantly (M = 11, SD = 11.8, 95% CI: [7.1, 14.9]), *t(34)* = –5.5, *p* < 0.001 (see Fig. 2). The effect size as measured by Cohen’s *d* was 1.12, indicating a very large effect size. Among the participants with uncontrolled hypertension at the start of the study (*n* = 30), 86.7% achieved BP control as defined by BP <140/90 or <130/80 mmHg with comorbid type 2 diabetes.

### Medication adherence

Preintervention, 28.6% (*n* = 10) of participants had low medication adherence, 37.1% (*n* = 13) had medium medication adherence, and 34.3% (*n* = 12) had high medication adherence.

**FIG. 3.**
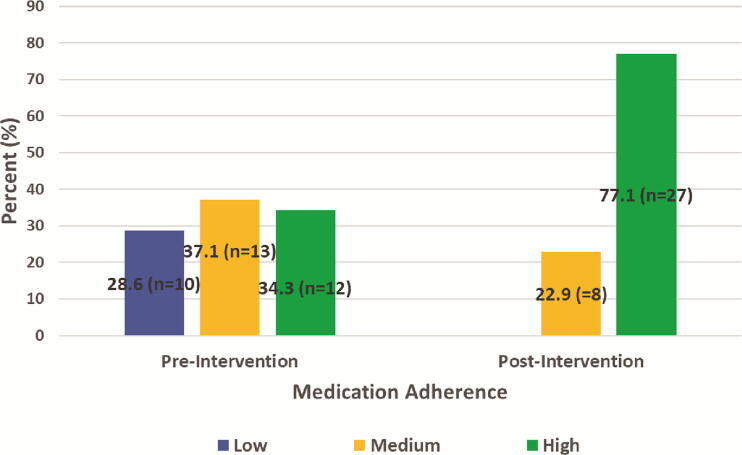
Medication adherence scores pre and postintervention. Preintervention, 28.6% (*n* = 10) of participants had low adherence, 37.1% (*n* = 13) had medium adherence, and 34.3% (*n* = 12) had high adherence. Postintervention, 22.9% (*n* = 8) of participants had medium adherence and 77.1% (*n* = 27) had high adherence; no participant had low adherence.

Postintervention, 22.9% (*n* = 8) of participants had medium medication adherence, and 77.1% (*n* = 27) of participants had high medication adherence. No participant had low medication adherence postintervention. The mean difference in medication adherence scores pre and postintervention improved significantly (M = 0.9, SD = 1.1, 95% CI: [0.52, 1.28]), *t*(34) = 5.0, *p* < 0.001 (see Fig. 3). The effect size, measured by Cohen’s *d*, was 0.82, indicating a large effect size.

### Hypertension knowledge

**FIG. 4.**
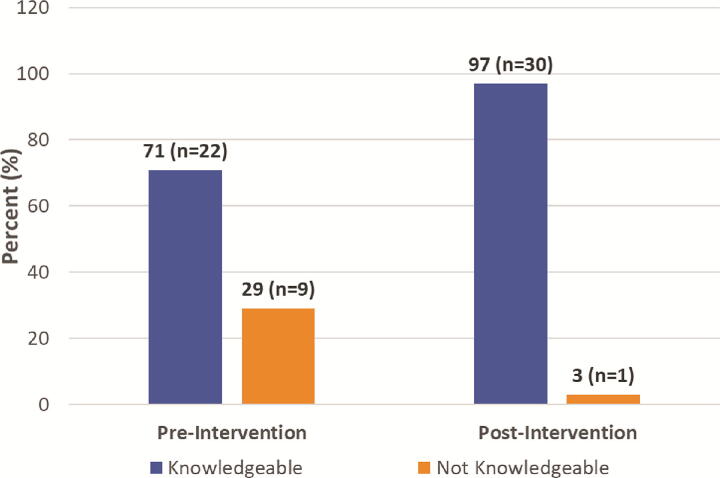
Hypertension knowledge test scores pre- and postintervention. Preintervention, 71% (*n* = 22) of participants scored ≥80% on the hypertension knowledge test. This increased to 97% (*n* = 30) postintervention.

Preintervention, 71% (*n* = 22) of our sample was knowledgeable as defined by a hypertension knowledge test score of 80% or greater. This improved to 97% (*n* = 30) postintervention. The average prehypertension knowledge score was 82.6% ± 9.3, and the average posthypertension knowledge score was 94.8% ± 5.6. The mean difference in hypertension knowledge test scores pre/postintervention improved significantly (M = 12.5, SD = 8.2, 95% CI: [9.7, 15.3]), *t(31)* = 8.4, *p* < 0.001 (see Fig. 4). The effect size, measured by Cohen’s *d*, was 1.52, indicating a large effect size.

### Feasibility

All 35 participants enrolled in this hypertension disparity study completed the program.

Adherence to daily BP monitoring was 89.8%, and the attendance rate at weekly phone coaching sessions was 94.7%. Most participants (*n* = 31) completed postintervention assessments except for four who did not complete the hypertension knowledge test. This resulted in 11.4% missing data. Provider adherence to the BP protocol was assessed through BP clinic volume, RPM enrollment, repeat BP documented in the chart, and the BP follow-up rate.

Six weeks after the hypertension practice protocol was implemented, BP clinic volume was at 50% of capacity, with a consistent increase in the number of visits per week. RPM enrollment doubled and repeated BP s documented in the EHR increased by 9-fold. In addition, there was a 7-fold increase in the number of patients with a scheduled BP follow-up. Overall adherence to the components of the BP protocol remained high at 6 months except for BP clinic volume; clinic visits started to decline around 3 months. Participants overwhelmingly expressed positive feedback toward the intervention throughout the study.

## Discussion

In the United States, Black Americans have the highest prevalence of hypertension,^1^ are 40% more likely to have uncontrolled BP,^2^ and are five times more likely to die from hypertension compared with non-Hispanic whites.^3,4^ The aim of this study was to translate effective strategies to address racial disparities in BP control at the local level through an intervention consisting of remote BP monitoring, phone coaching on hypertension lifestyle, medication intensification, and a hypertension protocol.

This quality improvement project demonstrated statistically significant improvements across all outcomes, including a notable reduction of 23 mmHg in SBP at 12 weeks. This reduction in SBP exceeds that demonstrated in other multicomponent intervention studies in which SBP reductions ranged from 9.7 to 21.6 mmHg at 3–6 months.^15–18^ Similarly, BP control as defined by BP <140/90 exceeded other studies in which BP control ranged from 64% to 81% at 4–6 months.^15,16,18^ Direct comparisons, however, should be avoided due to methodological differences. Most of the referenced studies were randomized controlled trials or quasiexperimental observational studies conducted in broader community settings with larger and more diverse sample populations. In contrast, our quality improvement initiative utilized a convenience sample within a single clinic setting and had a shorter follow-up period. Variation in study design, sample characteristics, and follow-up duration may have influenced the magnitude of our intervention’s effect, resulting in an overestimation of our intervention’s effectiveness.

Improvements were also observed in medication adherence and hypertension knowledge. All participants were on antihypertensive medication at enrollment; however, only 34% reported high medication adherence as defined by an MMAS score of 8. Self-reported medication adherence rose to 77% postintervention, with all participants reporting moderate to high medication adherence by program completion. Compared with an analogous quality improvement study conducted by Odemelam et al. in which phone coaching was combined with HBPM, our study achieved a greater improvement in medication adherence (+43% vs. +30%) despite our participants reporting less adherence to medication at baseline (66% vs. 54%).^17^ Both studies were similar in sample size and utilized convenience sampling; however, the Odemelam et al. study had a shorter follow-up period and focused on fewer intervention components. The observed differences in medication adherence may have resulted from the combined effects and longer duration of the interventions applied in our study.

Hypertension knowledge, as defined by a hypertension knowledge test score of 80% or greater, was moderately low at baseline (71%) and increased postintervention (97%).

Compared with Odemelam et al., our intervention had a lesser effect on hypertension knowledge (+26% vs. +45%) but resulted in comparable knowledge scores postintervention (97%).^17^ This may have resulted from baseline differences in education and health literacy in our study samples and differences in study design.

All participants completed the program. Most adhered to daily BP monitoring and attended weekly phone coaching sessions. We used telehealth technology, phone calls, and text messaging to avert poor attendance and completion rates noted across studies that involved on-site visits.^15,19–21^ Provider adherence to the BP protocol remained high except for BP clinic follow-up. This may have resulted from increased primary care physician follow-up and changes in clinic staffing. In addition, as RPM utilization increased, many providers were titrating medication outside of formal clinic visits, with patient communication limited to phone calls or portal messaging.

This quality improvement project demonstrates that BP, medication adherence, and hypertension knowledge among Black Americans can be improved through the combination of remote BP monitoring, phone coaching with culturally congruent care, medication intensification, and a hypertension practice protocol. This aligns with several studies that have demonstrated the potency of a combined approach on hypertension management in this population and offers a model for implementation in the primary care setting.^15,16,18,20,21^ Our study was not designed to assess the impact of the individual components of the intervention but suggests that weekly contact with a clinician may have engendered a sense of accountability, enhancing hypertension self-care behaviors and medication adherence. This high-touch intervention, incorporating daily reminders to check BP, text messaging to reinforce treatment plans, and hypertension education and weekly phone counseling, demonstrated the benefits of increased provider contact. The application of biofeedback through RPM allowed patients to see the effect of medication and hypertension lifestyle adherence on their BP in real-time, reinforcing hypertension self-care. In addition, discussing beliefs about hypertension, addressing medication aversion and side effects, and prescribing combination pills to reduce pill burden contributed to improved outcomes. Employing racial/ethnic congruence, psychoeducation and active listening during counseling fostered the development of trust, shared decision-making, and self-efficacy. These adjunctive biopsychosocial interventions increased health and social support, positively impacting our outcomes.

Our quality improvement project incorporated multiple components known to improve BP control in racial/ethnic minorities; however, the selected components and the application of the intervention were unique. This study embedded the intervention within a primary care practice by utilizing a nurse practitioner to extend the patient–provider relationship. Racial/ethnic and cultural congruence was employed in accordance with studies demonstrating effectiveness and improved health outcomes when applied among racial/ethnic minorities.^15,22^ This helped address cultural barriers to hypertension care. Our study is one of a few to employ direct access to a health care provider through two-way text communication, to exclusively target disparities in health outcomes in the primary care setting, and to intervene at the patient, provider, and organizational level. It is also one of a few studies to examine the use of telehealth in minority populations, using technology to facilitate the implementation of a potentially complex intervention and to extend clinical care. Several implementation challenges were encountered in this high-touch intervention including increased provider workload, limitations in reimbursement for phone coaching, and the organizational costs of supplying wireless BP monitors. While these challenges were manageable within this short-term quality improvement project, they are important considerations for long-term sustainability and broader implementation. Utilizing care management teams or CHWs can help alleviate provider workload and may offer alternative billing options for phone coaching.

In addition, partnering with device vendors and leveraging shared savings through value-based contracts could help offset the organizational costs of supplying remote monitoring equipment.

### Limitations

While this quality improvement project achieved relevant reductions in BP, the study design limits the ability to establish causality and rule out confounding factors such as medication adjustments and lifestyle changes that may have impacted our findings. This project was undertaken as part of the first author’s doctoral DNP/MPH Scholarly Project and was constrained to a quality improvement design, which did not allow for the inclusion of a control group. In addition, the duration of the project was limited to 12 weeks, restricting implementation and follow-up. Our small sample of predominantly older women limits generalizability and broader applicability to other demographic groups and does not represent the full diversity of Black individuals living with hypertension. As such, caution should be taken when applying these results to avoid overgeneralization. That said, this work remains meaningful as it addresses the needs of a group whose experiences and health challenges are frequently overlooked in both research and clinical practice. The study was conducted at a single site, which impacts potential scalability. Convenience sampling may have introduced sampling bias, with those who chose to participate more motivated to improve their health compared with the general population. This may have resulted in greater engagement with the intervention, potentially inflating the observed effectiveness. This limits the generalizability of the findings to less motivated or harder-to-reach populations and may not fully reflect the intervention’s impact in real-world settings. Although proper BP measurement technique was verified at the time of enrollment, we could not assure adherence to proper technique at home, which may have affected BP readings. Medication adherence was measured using self-reported data (MMAS-8), which is subject to recall and social desirability bias. In addition, questionnaires relied on self-report and may have been affected by language proficiency and response bias, which also limits our findings. Despite screening for eligibility through the EHR, 14.3% of participants had normal BP at enrollment. During recruitment, we targeted patients with BPs >140/90 at prior office visits; however, inaccuracy of in-office readings may have led to an overestimation of BP. Nevertheless, most of our sample had uncontrolled hypertension at enrollment, enabling us to demonstrate the efficacy of the intervention on BP control. Due to resource limitations, no follow-up measurements were taken to determine if the intervention effects persisted beyond the 12-week study period.

This limits our ability to determine the long-term impacts of the intervention. We also do not have data on the cost-effectiveness of the intervention, which potentially limits implementation of the intervention in the long term and in other settings.

## Conclusion

This study demonstrates the feasibility of translating an equity-centered intervention based on best available evidence and aimed at closing gaps in health in the primary care setting. Although other confounders cannot be ruled out, our findings suggest that targeting multilevel determinants affecting BP control among Black Americans may be effective in improving BP, medication adherence, and hypertension knowledge over 12 weeks. We could not ascertain whether these effects could be sustained beyond the study period. Future studies are needed to assess long-term outcomes, as well as the cost-effectiveness of the intervention. Further research is also needed on the efficacy of the intervention in diverse, larger primary care settings, among other racial/ethnic minority groups that experience similar disparities in BP control, and on other disease conditions that commonly accompany hypertension such as diabetes and obesity. To scale up this intervention, broader enrollment methods are necessary to avoid potential bias from convenience sampling and to enhance generalizability. In addition, adopting the intervention for broader health care settings including rural and resource-limited clinics is needed for scalability. This could involve leveraging nurses or CHWs to deliver hypertension care, incorporating mobile health technologies for education and substituting wireless monitors with more affordable alternatives. These adaptations could improve the feasibility and cost-effectiveness of the intervention across a variety of clinical environments, supporting the broader implementation of this intervention to help close inequities in hypertension control.

## Abbreviations Used

BP: blood pressure
CHWs: community health workers
CI: confidence interval
DBP: diastolic blood pressure
DNP/MPH: Doctor of Nursing Practice/ Master of Public Health
EHR: electronic health record
HBPM: home blood pressure monitoring
M: mean
MMAS: Medication Adherence Scale
PCP: primary care provider
RPM: remote patient monitoring
SBP: systolic blood pressure
SD: standard deviation
