# Supplementary figures and images for: Reducing Racial Disparities in Hypertension Control Using a Multicomponent, Equity-Centered Approach
Source: Health Equity. 2025 Aug 27;9(1):416–24. doi: 10.1177/24731242251371424 (PMC12412386; doi:10.1177/24731242251371424)

## Appendix A

### Work Breakdown Structure

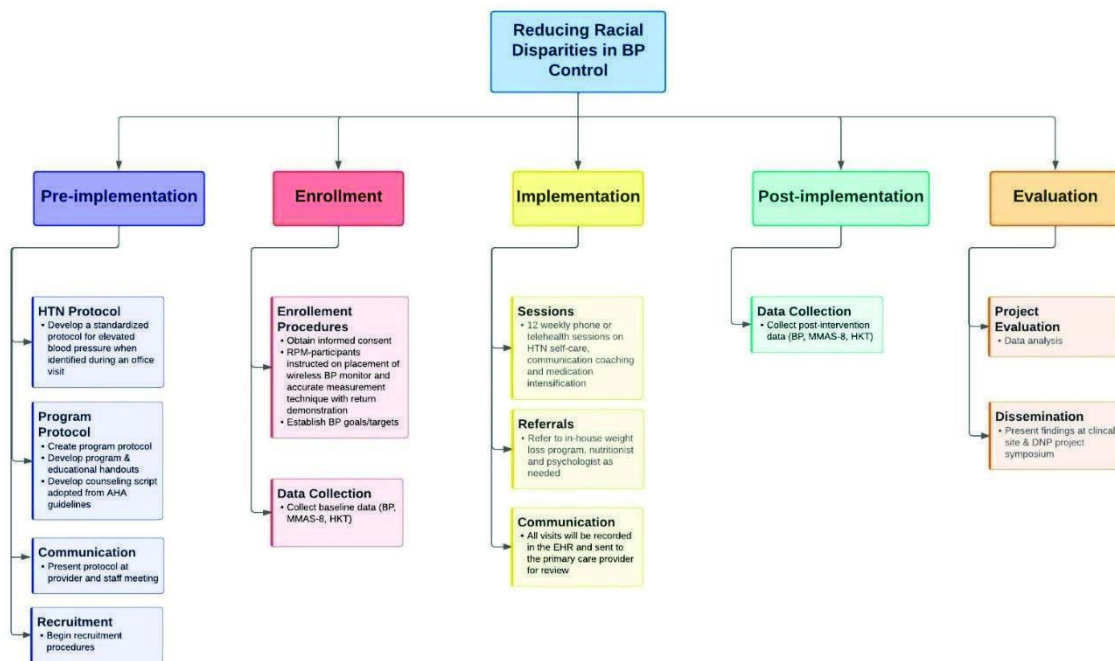

Appendix B

Hypertension Protocol

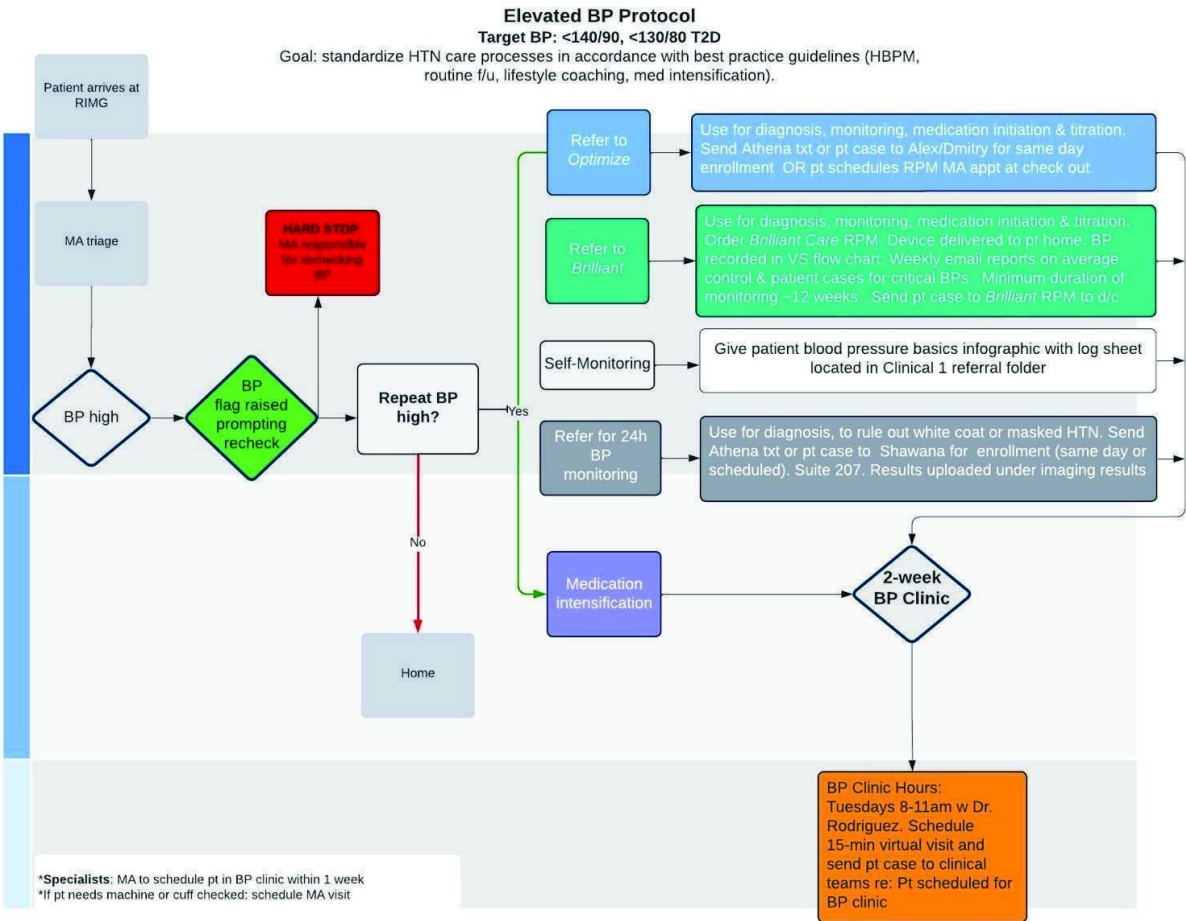

Supplement: Supplementary Appendix [file 24731242251371424_suppl_appendix.pdf]
